# Supplementary figures and images for: Differences in splicing defects between the grey and white matter in myotonic dystrophy type 1 patients
Source: PLoS One. 2020 May 14;15(5):e0224912. doi: 10.1371/journal.pone.0224912 (PMC7224547; doi:10.1371/journal.pone.0224912)

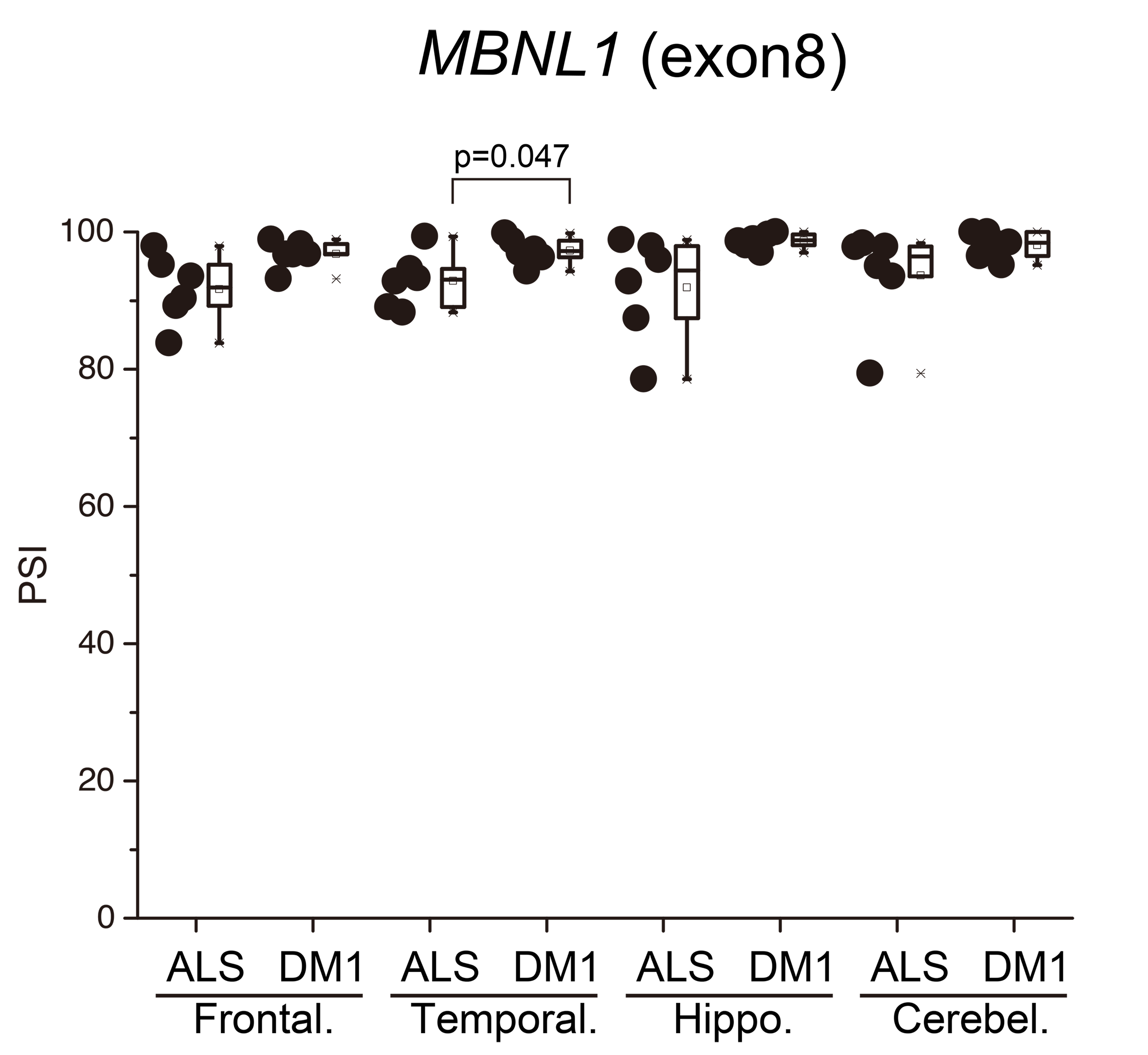

Supplement: S1 Fig — Inclusion ratios of splicing changes in several brain regions. PSI values of MBNL1 exon 8 was compared by Welch’s T-test. In box-and-whisker plot, the line inside the box is the median, square symbol is the average. ALS, amyotrophic lateral sclerosis; DM1, myotonic dystrophy type 1; Frontal., Frontal lobe; Temporal., Temporal lobe; Hippo., Hippocampus; Cerebel., Cerebellum. (TIF) [file pone.0224912.s001.tif]

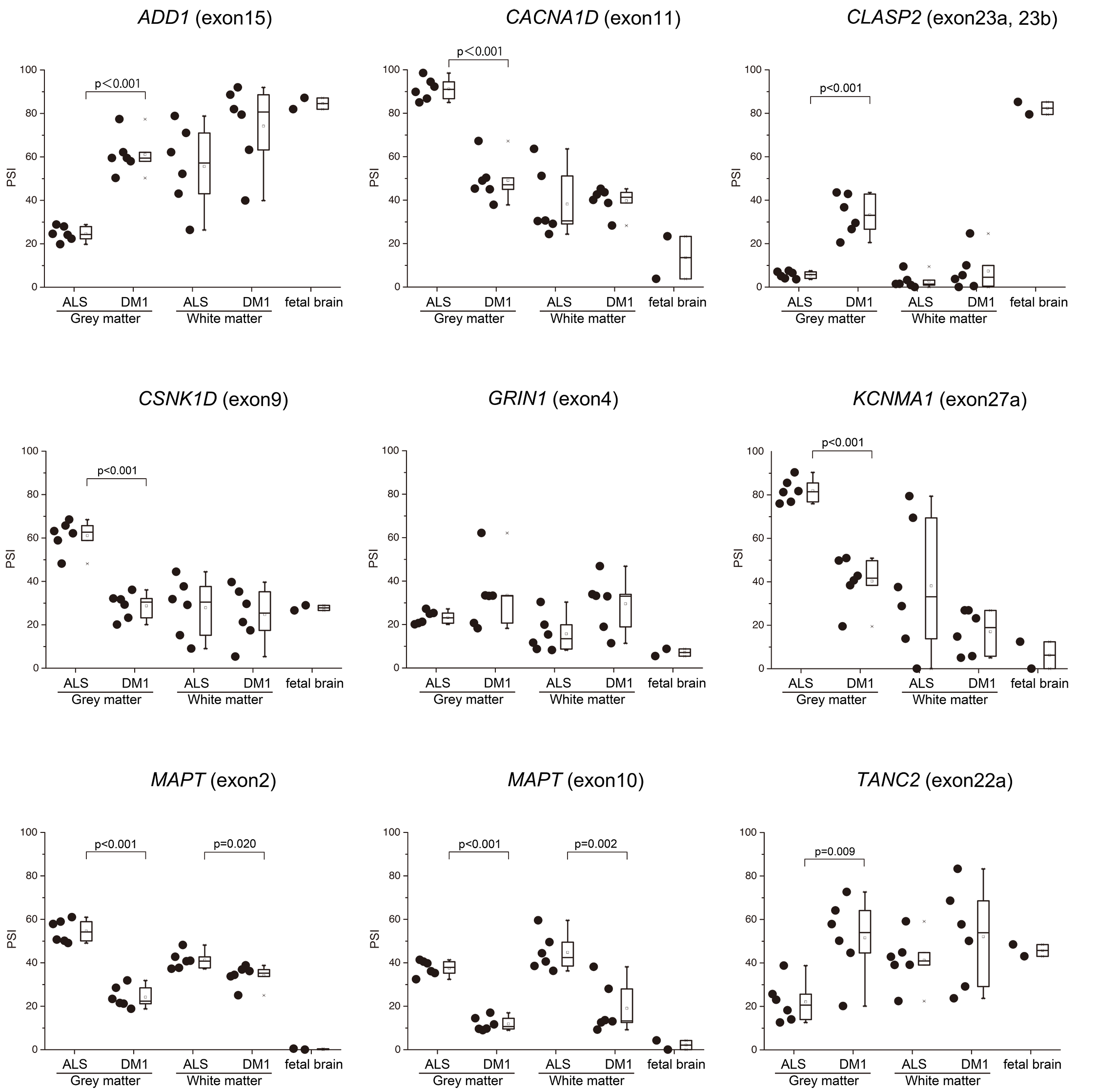

Supplement: S2 Fig — Inclusion ratios of splicing changes in the GM and WM. PSI values of all examined genes were compared by Welch’s T-test. In box-and-whisker plot, the line inside the box is the median, square symbol is the average. ALS, amyotrophic lateral sclerosis; DM1, myotonic dystrophy type 1. (TIF) [file pone.0224912.s002.tif]
