# Supplementary material for: Differences in splicing defects between the grey and white matter in myotonic dystrophy type 1 patients
Source: PLoS One. 2020 May 14;15(5):e0224912. doi: 10.1371/journal.pone.0224912 (PMC7224547; doi:10.1371/journal.pone.0224912)
Supplement: Attachment — Submitted filename: Response to Reviewers.docx [file pone.0224912.s003.docx]

Jan 31, 2020

Dr. Joerg Heber

Editor-in-Chief

*PLOS ONE*

Dear Editor:

Thank you for inviting us to submit a revised draft of our manuscript entitled, “Differences in splicing defects between grey and white matter in myotonic dystrophy type 1 patients.” to *PLOS ONE*. We also appreciate the time and effort you and each of the reviewers have dedicated to providing insightful feedback on ways to strengthen our paper. Thus, it is with great pleasure that we resubmit our article for further consideration. We have incorporated changes that reflect the detailed suggestions you have graciously provided. We also hope that our edits and the responses we provide below satisfactorily address all the issues and concerns you and the reviewers have noted.

To facilitate your review of our revisions, the following is a point-by-point response to the questions and comments delivered in your letter dated Nov 16 2019.

The paper was coauthored by Masamitsu Nishi, Masataka Igeta, Mitsuru Furuta, Koichi Suenaga, Tsuyoshi Matsumura, Harutoshi Fujimura, Kenji Jinnai, and Hiroo Yoshikawa. In this draft of our manuscript we added Dr. Igeta as a coauthor as he has newly contributed to the statistical analysis.

According to editor’s recomendation, we provided the original underlying images for all blot data at <http://www2.hyo-med.ac.jp/~ma-nishi/> .

Reviewer #1

Response: We thank the reviewer for his or her thoughtful and thorough review and believe the input has made our research more scientifically meaningful. We address all of the concerns here.

.

Abstract:

The abstract fails to summarise some important factual data reported by the authors. Instead the text is too speculative. It requires some careful re-writing.

**→The manuscript was reconsidered and refined.**

Factual mistakes:

The manuscript includes a few factual mistakes that must be corrected:

• Page 5, line 87: “Aberrant splicing of MBNL1 in the DM1 brain has been previously reported [23], but that of MBNL2 is still unknown.”

• Page 16, page 274: “however, we are the first to demonstrate MBNL2 exon 5 and exon 8 inclusion in the DM1 brain.”

MBNL2 missplicing in human brains has been previously reported in at least a couple of publications (Hernandez-Hernandez, 2013, Rare Diseases, 1: e25553; Sicot, 2017, Cell Reports, 19: 2718).

**→ I modified the manuscript that you pointed out, and added these papers in the reference. Missplicing of MBNL2 exon 5 (54nt) in human brains has been reported, but that of MBNL2 exon 8 is not reported.**

Statistical methods:

• The selection of the statistical tests is not convincingly justified. The Welch’s t-test used assumes normal distribution. Have the authors tested the normality of their data prior to the test?

**→We asked Dr. Igeta, a medical statistician of our college to review the statistical method and decided to include him as a co-author.**

**We performed the Shapiro-Wilk test for testing the normality. If the Shapiro-Wilk test was significant at least one group within a group comparison, we applied the Wilcoxon rank sum test. Then we observed the consistent results of the Welch’s t-test and Wilcoxon test for these cases.**

• In Figure 1 and Figure 3 the authors use a pairwise Welch’s t-test. It is unclear what has been “paired” in the analysis.

**→ “Pairwise” is a pair of the control and DM1 at each brain areas. It is also described in the manuscript. The test between the GM and WM in the control was omitted from the present results. Figure 3 is modified accordingly.**

• Welch’s t-test is known to perform poorly with low samples sizes. The authors should consider a more conservative non-parametric test.

**→The Wilcoxon rank sum test evaluates the location shift between groups assuming the same shape of data distributions. We considered the assumption of the Wilcoxon rank sum test does not necessarily fit to our data. For example, the PSI of MBNL1 (exon 5) at Hippocampus in Figure1 have different variances between groups, which indicate difference shape of distributions. We can see many of our data shows such distributions. Therefore, we showed the p^ values of the Welch’s t-test because there is no statistical test uniformly recommended to our data.**

Exon nomenclature:

The nomenclature of exons is vague and ambiguous, giving ample opportunity to confusion and misunderstanding. The discrepancy between databases makes it impossible today to identify an exon simply by their number, without reporting the reference genome/database used. To unequivocally identify the alternative exons investigated, the authors should provide their size and, ideally, their genomic coordinates.

**→We corrected MAPT exon number and added number of base pairs of all examined exons.**

Experimental conditions and rationale:

• Quantitative analysis of alternative splicing requires linear conditions of PCR amplification. The number of cycles used (36 and 42) seems exceptionally high. One wonders if they fall within the linear range of amplification. Given the extensive comparisons performed and the small magnitude of some of the differences reported, this is a critical point. Adequate controls must be included to demonstrate the linearity of the PCR amplification.

**→To determine the cycle number, we confirmed the linearity of the PCR amplification at 32 (for comparison among several brain regions) or 36 (for comparison between the GM and WM). Then we retried RT-PCR study at each cycle.**

• The rationale behind some experiments is not formulated and the implications of the conclusions are not properly outlined in the results section. E.g. western blot analysis of MBNL2 (page 15, lines 251 – 253). The relevance of this experiment is unknown, and the conclusions drawn are missing in the results section. Additionally, the variability between samples is very high making it very difficult to produce robust conclusions from the investigation of 3 patients and non-DM controls.
• There is an unjustified focus on the analysis of MBNL2 protein. In reality we do not know the distribution of MBNL1 and MBNL2 between GM and WM. The parallel analysis of MBNL1 protein levels (and eventually CELF proteins) would provide some useful insight and support to some of the hypotheses discussed in the final section of the paper.

**→ As you pointed out, we decided to omit the protein analysis from this post due to the problem of the number of sample.**

Data presentation and discussion:

• The text in page 13 (lines 219 – 222) does not match the data presented in Figure 3. The authors should review the inclusion and exclusion of some alternative cassettes.

**→We corrected the errors.**

• The data discussed on pages 13-14 (lines 230 – 236) is difficult to follow without a visual support (table or figure). More importantly, this data requires statistically testing to be convincing and to support the title of the paper.

**→We added a figure showing the average level of PSI change and Statistical tests were also performed (Figure 4).**

Typographic errors:

Overall the quality of the English is good, but there are a few minor typographic errors. E.g. Page 5, line 93: “…and the other genes that controlled by MBNL2”. The authors should carefully review their manuscript.

**→We corrected the typographic errors.**

Reviewer #2:

Response: We would like to thank the reviewer for his thoughtful comments and efforts towards improving our manuscript. We address all of the concerns here.

Major concerns:

1) Nothing is known about the control and DM1 patients

age, gender, duration of the disease, congitive status, type of DM1 ...

**→The age, gender, duration, and cognitive status of each sample were summarized in table 2.**

2) It is mandatory to know the stutus of the mutation in the brain tissue analysed using small-pool PCR. This will give the parental transmission, instability and min and maximal length of CUG expansion

3) make a correlation analysis between the mutation status and the intensity of the mis-splicing events

It will provide information about the sensivitity of the mis-splicing events with regards what is already described in the literature

**→From personal communication with Dr Nakamori, Osaka University, we know that the analysis of small pool PCR using brain samples is technically difficult. 　In addition, the editor suggested that “the characterization of the disease expansion size in tissue samples by small pool PCR is not a requisite to submit a revised version of the manuscript.” Taken together, we have determined not to carry out the analysis.**

4) Other transcripts should be include such as APP, Cacna1d.... APP is very important because the APP isoform 770 and 751 are expressed in the glial cells and not in neurons and therefore a mis-splicing of APP would suggest that the mutation is affecting glial cells as well and this has been scarcely documented (Goodwin et al., 2015).

**→ We confirmed that the isoform changes depending on the presence or absence of exon7 and 8 in APP. Then we made primers for exon 7 and 8 splicing and analyzed for APP splicing between GM and WM.**

5) Poly A selection has been described for MBNL2 in mouse brain, is it also true for the human brain?

**→We didn’t choose Poly A selection in this study.**

Minor concerns

Could authors use the numbering of exons as the appears in the litterature rather than as they appear in database. For instance, tau splicing is related to exon 2, 3 and 10 not to exon 12.

**→I corrected MAPT exon number and added number of base pairs of all examined exons.**
