# Supplementary material for: Differences in splicing defects between the grey and white matter in myotonic dystrophy type 1 patients
Source: PLoS One. 2020 May 14;15(5):e0224912. doi: 10.1371/journal.pone.0224912 (PMC7224547; doi:10.1371/journal.pone.0224912)
Supplement: Attachment — Submitted filename: Response to Reviewers.docx [file pone.0224912.s004.docx]

Response to reviewers

Reviewer #1: I commend the authors for the changes introduced in the revised version of the manuscript, and for their efforts to strengthen the quality of their work and the clarity of this report. As previously highlighted, the results reported by Kimura and colleagues are interesting. However, I still have some concerns that have not been convincingly resolved by the revision.

**>>** **We thank again the reviewer for his or her thoughtful and thorough review and believe the input has made our research more scientifically meaningful. We address all of the concerns here.**

Abstract

The second sentence is oversimplified and it gives the wrong idea that DM1 pathogenesis is explained by MBNL2 sequestration alone. We know that MBNL1 is also sequestered by RNA foci and plays a critical role, notably in the brain (Jiang et al. 2004; Hernandez-Hernandez et al. 2013, to cite but a few). This sentence should be modified.

**>> Thank you for your suggestion. We have incorporated your comments by changing MBNL2 to MBNL1 / 2 in the abstract.**

Introduction

Line 4. “It has been reported that fetal splice isoforms increase in adult DM1 tissues as a result of MBNL1/2 deletion”.

“Deletion” is not the most suitable word in this context. “Depletion” or “sequestration” would be more appropriate to describe the biological situation accurately.

**>>We have reflected this comment by correcting the word.**

Materials and methods

I am concerned that the ALS control samples used in this study may not be the most suitable to perform a careful comparison with DM1 brains. ALS tissue not only shows splicing deregulation [Nussbacher et al (2019) Neuron, 102: 294; Butti et al (2019) Frontiers in Genetics, 9: 712], but ALS brains may also display significant changes in white and grey matter [Zhou et al (2017) Mol Med Rep, 16: 4379; Turner et al (2015) Curr Neurol Neurosci Rep, 15: 45]. The authors should carefully address this point in their manuscript, and acknowledge its implications on the interpretation of results.

**>>Thank you for providing these insights. The "control" in the text has been corrected to “ALS”. In addition, limitations paragraph was set up to respond to the concerns using ALS as disease control.**

I think there is some sort of confusion in the use of paired and unpaired statistical tests. In Figure 1, when comparing PSI between control and DM1 samples, the authors claim to have used a paired Welch’s t-test. I do not understand the criteria used to pair subjects and I strongly believe this makes no biological sense. In my opinion the only situation where we could apply a paired Welch’s t-test would be the comparison of DPSI between GM and WM in Figure 4, in which we must pair GM and WM samples collected from the same individual. Surprisingly, legend of Figure 4 does not refer to the use of a paired statistical test.

**>>This is an important point. We deleted the term, “paired”, and its related description in the footnote of Figure 1 for correction. The statistical test which was actually applied in Figure1 was the (unpaired) Welch’s t-test as described in the section on “Statistical analysis”. Regarding to this correction, the footnotes of Figure 3, S1 and S2 were modified as well.**

**On the other hand, the footnote of Figure 4 related to the (unpaired) Welch’s t-test was not modified. We would like to explain more detail here about the statistical analysis for Figure 4, which was described as “The p-values for the difference between average ΔPSI GM and average ΔPSI WM ...” in the section on “Statistical analysis”. The definition of ΔPSI is “the average level of PSI change (ΔPSI), which was obtained by subtracting the average of PSI of the ALS from the DM1” as shown in the section on “Statistical analysis”. For example, ΔPSI GM are calculated as the difference of the averages of PSI of GM between groups (DM1-ALS). Furthermore, since there is no missing data in our PSI data, ΔPSI GM -ΔPSI WM, which was the basis for calculating the p-value in Figure 4, can be converted as follows:**

**ΔPSI GM - ΔPSI WM**

**= (average PSI of DM1 of GM - average PSI of ALS of GM)**

**- (average PSI of DM1 of WM - average PSI of ALS of WM)**

**= (average of PSI of (GM-WM) of DM1 - average PSI of (GM-WM) of ALS)**

**(GM-WM) in the above equation is a difference within a subject. However, the whole last equation expresses a comparison between DM1 and ALS groups. Therefore, we calculated the p-values in Figure 4 based on the (unpaired) Welch’s t-test for testing the difference between “average of PSI of (GM-WM) of DM1” and “average PSI of (GM-WM) of ALS” in the above equation.**

Results:

Figure 1 and Figure 3. The inclusion of the electrophoretic profiles is very useful and illustrates well the splicing defects reported in the graphs. However, it is unclear which exons are represented by each band. Proper labeling should be included, as it was done in the case of APP (exons 7 and 8) in Figure 3. The labelling is particularly important and relevant when multiple bands are detected for the same gene (more than 2), like in the analysis of MBNL2 (exon 8).

**>>We added the labels for exons7 and 8 of MBNL1 and MBNL2 in the figure 1.**

Page 14, line 225.

The authors write: “confirmed that the GM and WM were correctly separated”.

I believe this is an overstatement, and authors should tone it down the text, simply referring that samples were “enriched” for GM and WM.

**>>Thank you for your suggestion. We corrected the manuscript as you pointed out.**

Main text, page 14, line 226-227.

I find it hard to include TANC2 in the group of exon inclusion type in the fetal brain, since PSI for this alternative exon in this tissue is around 50% (if not slightly below). Are the authors referring to the adult brain? The sentence is vague, and the criteria to define exon inclusion version exon exclusion are not clearly defined.

**>>We agree with that TANC2 cannot be included in either pattern. We changed the patterns of AS in the fetal brain to three: 1) exon inclusion type (PSI ≥ 60%, ADD1, CLASP2, MBNL1/2), 2) exon exclusion type (PSI < 40%, APP, CACNA1D, CSNK1D, GRIN1, KCNMA1, MAPT) (Fig 3, S2 Fig), and 3) moderate type (40% ≤ PSI < 60%, TANC2). The manuscript and figures have been revised accordingly.**

Figure 3 and main text, page 14 lines 229-232.

The values in the text and the graph figure for MBNL1 (exon 5) do not match. In the text we read that PSI for MBNL1 exon 5 in the GM of DM1 patients is 57.43%. If we examine the graph carefully, the mean PSI represented is above 60% for the same tissue in the same group of subjects. The authors should correct this problem and review all the graphs carefully.

**>>You have raised an important query. In our box-and-whisker plot, the line inside the box is the median, square symbol is the average. We believe that the graph has correctly shown that the average PSI for MBNL1 exon5 in the GM of DM1 patients is 57.43%. To make this point clear, we added the sentence in the figure legends explaining that the average value is represented by square symbol in the box-and-whisker plot.**

Figure 4.

The p values of some comparisons are not included in the figure, so one assumes the difference is not statistically different. E.g. TANC2 exon 22a. However, it is confusing that simply by looking at the graph, the average DPSI represented for grey and white matter appears to be considerably different. To overcome this apparent inconsistency the authors should either represent SEM or plot the individual values around the average calculated.

**>>This is an important perspective. We added summary statistics such as SEM and 95% confidence interval in Figure 4.**

Discussion

Page 17, lines 281-289.

The authors elaborate on the splicing defects of GRIN1 in the hippocampus. Although interesting, two considerations should be highlighted in the discussion. The extent of the splicing defect relative to ALS patients (in other words, the value of DPSI) is very low, and the functional consequences of such a minor difference might also be minimal. Regardless of this limitation, the splicing defect of GRIN1 in the hippocampus does not represent a return to an embryonic splicing profile, and this is very interesting per se and should be further stressed.

**>> Thank you for your suggestions. We have inserted sentences that emphasizes the two considerations you pointed out.**

-------------------------------------------------------------------------------------------------------------------------

Reviewer #2: In this revised manuscript authors has addressed almost completely all concerns except for the size of CTG expansion. Thus, their diagnosis were mainly based on the disease onset.

**>>** **We would like to thank again the reviewer for his thoughtful comments and efforts towards improving our manuscript. We address all of the concerns here.**

However, as now indicated in table DM1 patient include early, juvenile, adult and late onset cases. Even if the number of sample is low, it would have been of interest to determine whether the amplitude of mis-splicing as the difference between grey and white matter is observed in each sub-type of the disease. This is in the scope of this paper since the mechanism underlying the mis-splicing is different between early or juvenile and adult / late onset. Large expansion are transmitted in the early and juvenile form whereas large expansion are acquired with ageing in adult and late onset DM1 case.

**>>Thank you for providing these insights. We agree with that it is important to determine whether the degree of mis-splicing, as the difference between the GM and WM, is related to each type of DM1 and the degree of cognitive decline. However, we could not determine a clear trend in this study, probably because the sample size was small and the variability of the PSI values between samples was large. We mentioned this point in the paragraph of Limitations.**

More importantly, authors have referred ALS brain tissue as a "control" tissue. The cause of ALS is not known and it is well-known that a mis-transport of ARN is instrumental of ALS. Please refer to ALS rather than to control brain tissue.

**>>Thank you for your suggestion. The "control" in the text has been corrected to “ALS”. In addition, limitations paragraph was set up to respond to the concerns using ALS as disease control.**
