# Supplementary material for: Differences in splicing defects between the grey and white matter in myotonic dystrophy type 1 patients
Source: PLoS One. 2020 May 14;15(5):e0224912. doi: 10.1371/journal.pone.0224912 (PMC7224547; doi:10.1371/journal.pone.0224912)
Supplement: Attachment — Submitted filename: Response to Reviewers.docx [file pone.0224912.s005.docx]

Response to reviewers

Reviewer #1: The authors have done a very good job clarifying some of the points, and strengthening the quality and impact of their manuscript.

I would like to point out a couple of minor details and bring up point for discussion.
**>>** **We would like to thank again the reviewer for his or her thoughtful comments and efforts towards improving our manuscript. We address all of the concerns here.**

1. Introduction, line 51-52.
It should rather be: “… such as muscleblind-like (MBNL) proteins”
**>> We agree with you. We have corrected the manuscript accordingly.**

2. Materials and Methods, line 140.
As discussed, and changed elsewhere in the manuscript, I would suggest: “To ensure enrichment of the GM and WM, …”
**>> Thank you for your suggestion. We have modified the manuscript accordingly.**

3. Figure 4.
Would it be possible to introduce error bars directly on the graph, representing the SEM values? The change would certainly make the figure clearer.

**>>Thank you for your suggestion. We have added error bars representing the SEM values of each Delta PSI on Figure 4.**

4.Figure 4 (suggestion for the authors’ consideration).
The authors explain the rationale behind the statistics performed, and the selection of the unpaired Welch’s t-test. Point taken.
Biologically, it would make sense to compare the Delta-PSI of an exon between the GM and WM of the same individual. In other words, when looking at the entire patient set, a paired test should compare the Delta-PSI in GM and Delta-PSI in WM by matching the GM and WM PSI values for the same patient.
How to go about and calculate the Delta PSI per exon, per individual DM1 patient? The solution would be to use as reference value the mean/median PSI for each exon among the ALS disease controls.
My feeling is that such paired statistical analysis could bring up significant differences between GM and WM that are otherwise confounded by inter-individual variability.
I leave this point to the authors’ consideration.

**>>** **Thank you for an interesting proposal about a paired statistical analysis. We agree that the paired test using reference value would provide more significant results.** **However, we have to recognize the paired analysis would require an important assumption: the adequacy on the reference value. Although the variance of PSI values in the ALS disease controls was smaller than that in DM1 patients, we think non-negligible variability was left in the mean or median PSI in the ALS subjects because it was calculated from only 6 subjects. Thus, we considered that it is difficult to explain the adequacy of taking the sample mean or median as the reference value.** **Therefore, we would like to hold our interpretation of the difference between GM and WM based on the current analyses.**
